# Health-related quality of life and physical activity collected via mobile application and wearable device in patients with HR +/HER2 − advanced breast cancer treated with palbociclib plus endocrine therapy or endocrine therapy alone: 6-month longitudinal study (JBCRG-26)

**DOI:** 10.1007/s12282-025-01744-0

**Published:** 2025-07-18

**Authors:** Hiroko Bando, Aya Ueda, Kaori Terata, Mihoko Doi, Shigenori E. Nagai, Masaya Hattori, Kenichi Watanabe, Nobuko Tamura, Manabu Futamura, Kei Koizumi, Naoki Niikura, Tempei Miyaji, Yasuaki Muramatsu, Linghua Xu, Norikazu Masuda, Shigehira Saji

**Affiliations:** 1 Department of Breast-Thyroid-Endocrine Surgery, Institute of Medicine, University of Tsukuba, Tsukuba, Japan; 2 Department of Breast-Thyroid-Endocrine Surgery, University of Tsukuba Hospital, Tsukuba, Japan; 3 Department of Breast and Endocrine Surgery, Akita University Hospital, Akita, Japan; 4 Department of Clinical Oncology, Hiroshima Prefectural Hospital, Hiroshima, Japan; 5 Division of Breast Oncology, Saitama Cancer Center, Saitama, Japan; 6 Department of Breast Oncology, Aichi Cancer Center Hospital, Aichi, Japan; 7 Department of Breast Surgery, Hokkaido Cancer Center, Hokkaido, Japan; 8 Department of Breast and Endocrine Surgery, Toranomon Hospital, Tokyo, Japan; 9 Department of Breast Surgery, Gifu University Hospital, Gifu, Japan; 10 Department of Breast Surgery, Hamamatsu University Hospital, Hamamatsu, Japan; 11 Department of Breast Oncology, Tokai University School of Medicine, Kanagawa, Japan; 12 Meaningful Outcome Consulting Inc, Tokyo, Japan; 13 Oncology Medical Affairs, Pfizer Japan Inc, Tokyo, Japan; 14 Access & Value, Pfizer Japan Inc, Tokyo, Japan; 15 Department of Breast Surgery, Graduate School of Medicine, Kyoto University, Kyoto, Japan; 16 Department of Medical Oncology, Fukushima Medical University, Fukushima, Japan

**Keywords:** Advanced breast cancer, Endocrine therapy, Health-related quality of life, HR +/HER2 −, Palbociclib, Physical activity

## Abstract

**Objectives:**

To summarize descriptively health-related quality of life (HRQOL) and physical activity (PA) evaluated with a mobile application and wearable device among patients with hormone receptor-positive/human epidermal growth factor receptor 2-negative (HR + /HER2 −) advanced breast cancer (ABC) treated with first- or second-line palbociclib plus endocrine therapy (ET) or ET alone.

**Methods:**

HRQOL was assessed with the EORTC QLQ-C30 at baseline and Day 15 of 6 treatment cycles (~ 24 weeks). PA metrics were averaged on a weekly basis for 24 weeks. Co-primary endpoints were mean change from baseline in Global Health Status (GHS) and sedentary time.

**Results:**

Ninety-nine patients were enrolled; 78 received palbociclib plus ET (mean age: 57.2 years; 75.6% initiated first-line treatment) and 21 received ET alone (mean age: 56.3 years; 90.5% initiated first-line treatment). Baseline mean GHS score was 60.9 in the palbociclib plus ET group and 64.3 in the ET alone group; mean changes from baseline to Day 15 of Cycle 6 were +4.8 and +2.9, respectively, and not deteriorated beyond the 10-point clinically significant threshold in either treatment group. Baseline mean sedentary time was 581 min/day in the palbociclib plus ET group and 513 min/day in the ET alone group; mean changes from baseline to Week 24 were −22 and −102 min/day, respectively.

**Conclusions:**

In this real-world study of women with HR+/HER2− ABC in Japan, neither palbociclib plus ET nor ET alone had any substantial detrimental impacts on HRQOL, according to patients’ assessments recorded in a smartphone-based mobile application, and PA, as measured by a wearable device.

**Trial registration:**

ClinicalTrials.gov NCT04736576; registered, February 3, 2021.

**Supplementary Information:**

The online version contains supplementary material available at 10.1007/s12282-025-01744-0.

## Introduction

Breast cancer (BC) incidence has been on the rise in Japan and is now the most common type of cancer among Japanese women with 97,142 new cases diagnosed in 2019 [1]. Approximately 5–10% of patients with BC are diagnosed with de novo advanced or metastatic BC (ABC) and 20–30% of patients diagnosed with early-stage BC will develop ABC over time [2]. Current guidelines recommend a cyclin-dependent kinase (CDK) 4/6 inhibitor in combination with endocrine therapy (ET) as the first-line treatment for hormone receptor-positive/human epidermal growth factor receptor 2-negative (HR+/HER2−) ABC, the most common subtype of ABC [3–5].

In Japan, palbociclib, the first-in-class CDK4/6 inhibitor, was approved in 2017 for the treatment of inoperable or recurrent BC [6]. The safety and efficacy of palbociclib plus ET were investigated in two pivotal trials (PALOMA-2 and PALOMA-3), in which palbociclib plus ET significantly prolonged progression-free survival (PFS) compared with placebo plus ET, and numerically, but not statistically significantly, extended overall survival [7–10]. Subgroup analyses of these trials also demonstrated palbociclib plus ET was effective in Japanese patients [11, 12].

With newer treatments and extended real-world survival of patients with BC [13], it is an important care priority to maintain and/or improve health-related quality of life (HRQOL). Improving HRQOL of patients with ABC and balancing it with survival is one of the ABC Global Charter goals [14–16]. In the PALOMA trials, multiple validated paper-based patient-reported outcome (PRO) assessment tools were utilized, and patients with HR +/HER2 − ABC who received palbociclib plus ET were found to maintain or improve their HRQOL relative to those who received ET alone [17, 18].

Another common assessment using self-reported questionnaires is physical activity (PA), which with ongoing technological advancements can now be more systematically and objectively measured among patients with cancer with wearable activity monitors [19]. PA, such as aerobic exercise, has been shown to improve functional status in patients with BC [20], while moderate and high intensity PA, both pre- and post-diagnosis, have been associated with significantly reduced risk of BC-specific mortality [21].

Some studies have evaluated the effects of palbociclib plus ET on HRQOL and daily activities of patients in real-world settings in Western populations [22–24]; however, such studies in Asian countries are limited. Furthermore, there is sparse information on HRQOL and PA among patients treated either with palbociclib plus ET or ET monotherapy in real-world settings in Asian countries. Therefore, we aimed to summarize descriptively changes in HRQOL and PA evaluated with a mobile application and wearable device among patients with HR +/HER2 − ABC treated with first- or second-line palbociclib plus ET or ET alone in Japan.

## Patients and methods

### Study design

This was a prospective, observational, multicenter study conducted across multiple sites in Japan (NCT04736576). Site investigators obtained Institutional Review Board (IRB)/Independent Ethics Committee (IEC) approval, screened patients for eligibility, obtained written informed consent of patients prior to study participation and enrolled patients (target enrollment: 100 patients). The study was conducted in accordance with the Ethical Guidelines for Medical and Health Research Involving Human Subjects issued by the Ministry of Health, Labour and Welfare and the Declaration of Helsinki.

### Patients

Adult female patients ≥ 20 years of age diagnosed with HR +/HER2 − BC with evidence of advanced or metastatic disease, initiating first- or second-line treatment, and with an Eastern Cooperative Oncology Group performance status (ECOG PS) of 0 or 1 were enrolled between February 2021 and September 2022. Patients were required to have ownership or regular access to an Apple iPhone or Android phone and willingness and ability to complete collection of data and to wear the wearable device for approximately 6 months. Patients were excluded from the study if they had malignancies other than ABC, were participating in any interventional clinical trial, and if they experienced large fluctuations in PA on a weekly basis (eg, shift workers).

### Treatment

Based on the discretion of treating physicians in routine clinical practice, patients received either palbociclib plus ET or ET alone (eg, letrozole, anastrozole, exemestane, fulvestrant, tamoxifen, toremifene). The observation period included 6 cycles of treatment (24 weeks); this timeframe was chosen since the incidence of adverse events (AE) related to palbociclib plus ET peaks within the first 6 months of treatment [25]. In the palbociclib plus ET group, palbociclib was administered for 3 consecutive weeks followed by 1 week off palbociclib treatment so that 1 cycle was equivalent to 4 weeks. If palbociclib treatment was temporarily interrupted (eg, due to an AE) and the start of the next cycle was delayed, the duration of 1 cycle may have exceeded 4 weeks, and the total observation period may have gone beyond 24 weeks. In the ET-alone group, 1 cycle was defined as 4 weeks. The end of the study period was defined as the end of the 6th cycle of initiated treatment. If patients discontinued the initiated treatment before completion of 6 treatment cycles (eg, due to disease progression) and switched to another treatment, the end of study was at the end of 24 weeks after the start of initial treatment. Participation in this study was not intended to change the routine treatment that patients received as determined by their prescribing clinicians.

### Patient-reported outcomes

A primary endpoint of this study was the change from baseline in the European Organisation for Research and Treatment of Cancer Quality-of-Life Questionnaire-C30 (EORTC QLQ-C30) Global Health Status (GHS) score. The EORTC QLQ-C30 was developed specifically for patients with cancer and has been utilized in clinical trials of palbociclib to understand the impacts on HRQOL and daily activities [18, 26]. The questionnaire employs 28 4-point Likert scales with responses from “not at all” to “very much” and two 7-point Likert scales for GHS. Responses to all items are then converted to a 0–100 scale. The linguistic and psychometric validation of the questionnaire into Japanese has been conducted and the Japanese translated version was utilized [27].

Secondary PRO endpoints included the 5 functional (physical, role, social, emotional, and cognitive) and 9 symptom (fatigue, nausea and vomiting, pain, dyspnea, insomnia, appetite loss, constipation, diarrhea, and financial difficulties) subscales of the EORTC QLQ-C30. Patients downloaded a smartphone-based mobile application for PRO assessments (ie, ePRO system) and were provided access to and trained on the use of the application. Patients completed questionnaire at baseline and on Day 15 of each cycle for 6 cycles via the ePRO system; ePRO data were subsequently transferred to the server hosted by the ePRO vendor (Signant Health; USA).

### PA metrics

Another primary endpoint of this study was change from baseline in the “sedentary time” of the PA metrics. PA metric data were collected via the wearable device (CentrePoint Insight Watch®, ActiGraph LLC, USA) and summarized at weekly intervals. Patients were provided with the device and instructed to wear it at all times during the baseline period (≥ 4 days prior to treatment initiation was recommended) and throughout the 6 treatment cycles, except while bathing and sleeping. Wear time of the device and other PA metrics captured included sedentary time, moderate-to-vigorous PA time, steps, light PA time, and calories. PA metrics were calculated according to algorithms reported by Staudenmayer and colleagues [28]. Patients were asked to bring the device worn in the past period at regular clinical visits occurring at approximately 30 day intervals; study site staff uploaded the PA data through the Actigraph application to the Actigraph cloud system.

### Other study measurements

Patient data, including baseline demographic and clinical characteristics, treatment and AEs were collected via electronic case report forms (Oracle Corporation; USA) by site investigators.

### Statistical analyses

GHS, functional and symptom subscale scores, PA metrics, and other patient data were summarized descriptively. Mean, median, ranges, and standard deviations (SDs) were reported for continuous variables and counts and percentage distributions for categorical variables. For GHS and functional subscales, higher scores indicate a better level of HRQOL and functioning, while for symptom subscales, a higher score indicates greater symptom severity. Mean (SD) changes from baseline in GHS recorded at Day 15 of each cycle were reported. Mean changes from baseline to Day 15 of Cycle 6 were also reported for functional and symptom subscales. A 10-point change from baseline was considered clinically significant [18, 29]. PA metrics were averaged over a week, which included ≥ 5 days of wearing the device for ≥ 10 h/day and were reported at baseline and each week for 24 weeks. Mean [SD] weekly changes from baseline in sedentary time were reported, as well as mean changes from baseline to Week 24 for the other evaluated PA metrics. Missing values were not imputed in this study and no formal hypothesis testing or statistical comparisons were planned or conducted.

## Results

### Patients

A total of 99 patients were enrolled; 78 received palbociclib plus ET and 21 received ET alone. Baseline patient demographic and clinical characteristics of the study groups are shown in Table 1**.** Among the patients who received palbociclib plus ET, mean age was 57.2 years, 75.6% initiated first-line treatment and 51.3% had visceral metastases. Among the patients who received ET alone, mean age was 56.3 years, 90.5% initiated first-line treatment and 33.3% had visceral metastases.

**Table 1:** Baseline patient demographic and clinical characteristics of the study groups

	Palbociclib + ET (n = 78)	ET alone (n = 21)
**Age, years**		
Mean (SD)	57.2 (10.0)	56.3 (10.1)
Median (range)	56.0 (35, 83)	52.0 (44, 75)
**Age group, years, n (%)**		
20 − < 45	6 (7.7)	1 (4.8)
45 − < 55	28 (35.9)	12 (57.1)
55 − < 65	26 (33.3)	1 (4.8)
≥ 65	18 (23.1)	7 (33.3)
**Body** **mass index, kg/m**^**2**^		
Mean (SD)	24.5 (5.5)	25.1 (5.0)
Median (range)	22.9 (16.9, 45.9)	24.1 (17.0, 35.0)
**Education**,^a^ **n (%)**		
Less than high school	5 (6.4)	1 (4.8)
High school diploma or equivalent	28 (35.9)	11 (52.4)
Some college	29 (37.2)	4 (19.0)
College degree or higher	15 (19.2)	5 (23.8)
**Employment,**^**a**^** n (%)**		
Full-time	28 (35.9)	7 (33.3)
Part-time	22 (28.2)	5 (23.8)
Currently retired/not employed for other reason	27 (34.6)	9 (42.9)
**Menopausal status,** **n** (%)		
Premenopausal	27 (34.6)	10 (47.6)
Postmenopausal	51 (65.4)	11 (52.4)
**ECOG PS, n (%)**		
0	67 (85.9)	18 (85.7)
1	11 (14.1)	3 (14.3)
**Line of therapy, n (%)**		
First	59 (75.6)	19 (90.5)
Second	19 (24.4)	2 (9.5)
**Stage at initial diagnosis, n (%)**		
I	11 (14.1)	1 (4.8)
IIA	15 (19.2)	7 (33.3)
IIB	3 (3.8)	1 (4.8)
IIIA	4 (5.1)	1 (4.8)
IIIB	6 (7.7)	1 (4.8)
IIIC	7 (9.0)	0
IV	25 (32.1)	9 (42.9)
Unknown	7 (9.0)	1 (4.8)
**Visceral metastases, n (%)**	40 (51.3)	7 (33.3)
**Bone-only metastasis, n (%)**	3 (3.8)	0
**Disease-free interval,**^**b**^ **n (%)**		
**With surgery**	52 (66.7)	12 (57.1)
< 24 months	7 (9.0)	1 (4.8)
≥ 24 months	45 (57.7)	11 (52.4)
**Treatment-free interva**l**,**^**c**^** n (%)**		
**With adjuvant therapy**	49 (62.8)	11 (52.4)
< 12 months	25 (32.1)	7 (33.3)
≥ 12 months	24 (30.8)	4 (19.0)

*ECOG PS* Eastern cooperative oncology group performance status, *ET* Endocrine therapy, *SD* Standard deviation

^a^There was one patient missing education and employment data in the palbociclib + ET group

^b^Disease-free interval was defined as the period from date of surgery to date of recurrence/metastasis

^c^Treatment-free interval was defined as the period from date of end of adjuvant therapy to date of recurrence/metastasis

Seventy-four of 78 patients (95%) who received palbociclib plus ET completed the 6-cycle observation period; 68 of 74 patients completed both the 6-cycle treatment and observation and 6 of 74 patients discontinued treatment but completed the observation period with receiving ≥ 1 follow-up cancer therapy. Among the 21 patients who received ET alone, 20 (95%) completed the 6-cycle observation period; 17 of 20 patients completed both the 6-cycle treatment and observation; and 3 of 20 patients discontinued treatment but completed the observation period with receiving ≥ 1 follow-up cancer therapy**.**

### HRQOL

**Fig. 1:**
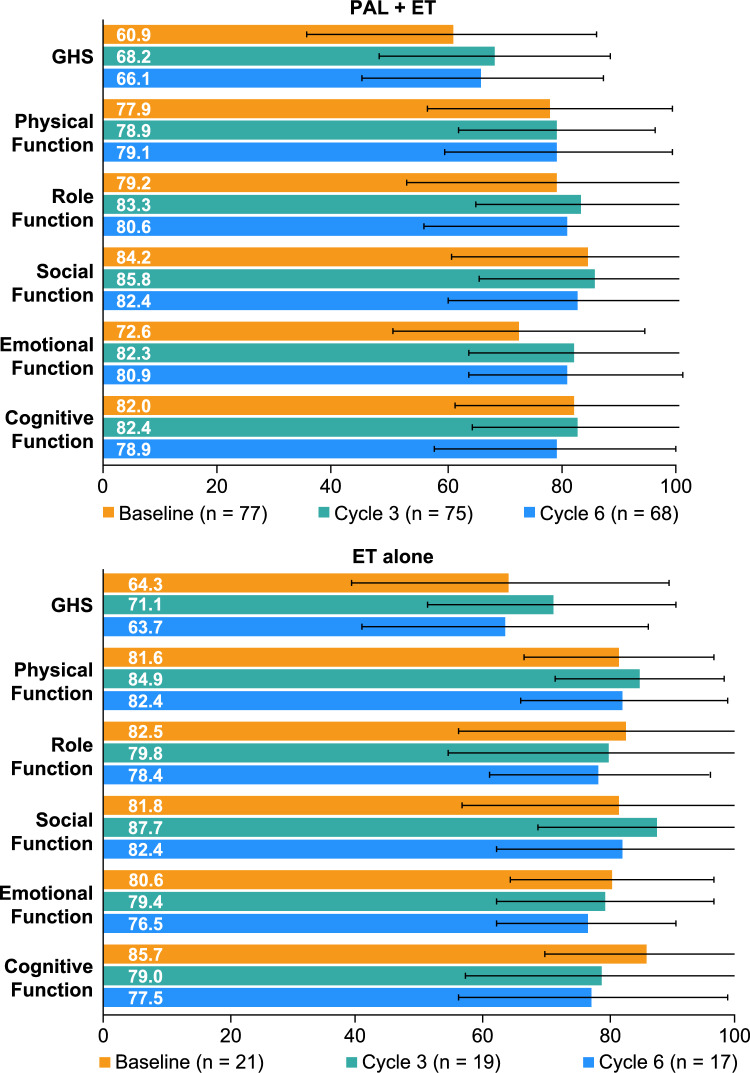
Mean (SD) scores of EORTC QLQ-C30 GHS and functional subscales. *EORTC QLQ-C30* European organisation for research and treatment of cancer quality-of-life questionnaire-C30, *ET* Endocrine therapy, *GHS* Global health status, *PAL* Palbociclib, *SD* Standard deviation. Higher scores indicate a better level of HRQOL and functioning

**Fig. 2:**
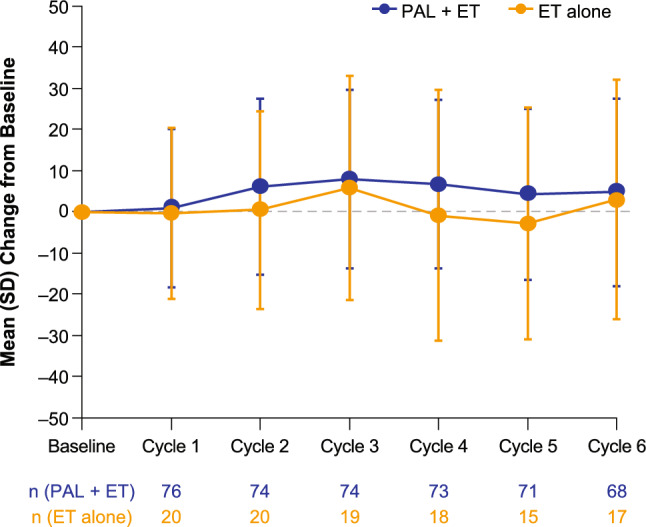
Mean change from baseline in GHS score. *ET* Endocrine therapy, *GHS* Global health status, *PAL* Palbociclib, *SD* Standard deviation. Higher scores indicate better GHS

Baseline mean (SD) GHS score was 60.9 (25.2) in the palbociclib plus ET group and 64.3 (25.0) in the ET alone group (Fig. 1). At Day 15 of Cycles 3 and 6, mean (SD) scores were 68.2 (20.2) and 66.1 (20.9), respectively, in the palbociclib plus ET group and 71.1 (19.7) and 63.7 (22.6), respectively, in the ET alone group (Fig. 1). The mean (SD) changes in GHS score from baseline to Day 15 of Cycles 3 and 6 were 8.0 (21.6) and 4.8 (22.8), respectively, among patients who received palbociclib plus ET and 5.7 (27.2) and 2.9 (29.0), respectively, among those who received ET alone and were not deteriorated beyond the 10-point clinically significant threshold in either treatment group (Fig. 2).

Mean functional subscale scores were also maintained throughout the 6-cycle observation period (Fig. 1). For the palbociclib plus ET group, mean changes in scores from baseline to Cycle 6 for physical (0.4), role (1.0), social (−2.5), emotional (9.3), and cognitive (−3.2) functioning were not deteriorated (Fig. 1). Likewise, for the ET alone group, mean changes in scores from baseline to Cycle 6 for physical (−1.2), role (−2.9), social (2.0), emotional (−3.4), and cognitive (−7.8) functioning were not deteriorated (Fig. 1).

Mean symptom subscale scores were generally maintained throughout the 6-cycle observation period; however, large variations were observed within study groups (Online Resource Fig. 1). For the palbociclib plus ET group, mean changes in scores from baseline to cycle 6 for symptoms of fatigue (−0.5), nausea and vomiting (−3.4), pain (0), dyspnea (−0.5), insomnia (−3.4), appetite loss (−6.9), constipation (5.9), diarrhea (−3.4), and financial difficulties (3.4) were not deteriorated (Online Resource Fig. 1). For the ET alone group, mean changes in scores from baseline to cycle 6 for symptoms of fatigue (−1.3), nausea and vomiting (−5.9), pain (2.9), dyspnea (−3.9), insomnia (5.9), appetite loss (−3.9), constipation (5.9), diarrhea (2.0), and financial difficulties (−9.8) were not deteriorated (Online Resource Fig. 1).

### Physical activity

At Week 24, 93% of patients in the palbociclib plus ET group and 82% in the ET alone group continued wearing their wearable device. Over the 24-week observation period, mean wear time ranged from 926 to 976 min/day among those who received palbociclib plus ET and from 842 to 912 min/day among those who received ET alone (Online Resource Fig. 2).

**Fig. 3:**
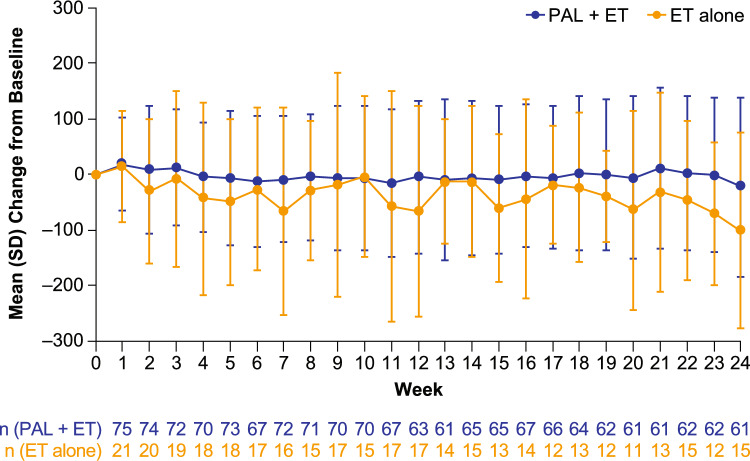
Mean weekly changes from baseline in sedentary time. *ET* Endocrine therapy, *PAL* Palbociclib, *SD* Standard deviation

Baseline mean (SD) sedentary time (averaged between enrollment day and the day before treatment initiation) was 581 (179) min/day in the palbociclib plus ET group and 513 (99) min/day in the ET alone group. In Week 24, sedentary time averaged 550 (197) min/day in the palbociclib plus ET group and 421 (118) min/day in the ET alone group. Mean (SD) change in sedentary time from baseline to Week 24 was − 22 (161) min/day among patients who received palbociclib plus ET and − 102 (176) min/day among those who received ET alone (Fig. 3).

**Fig. 4:**
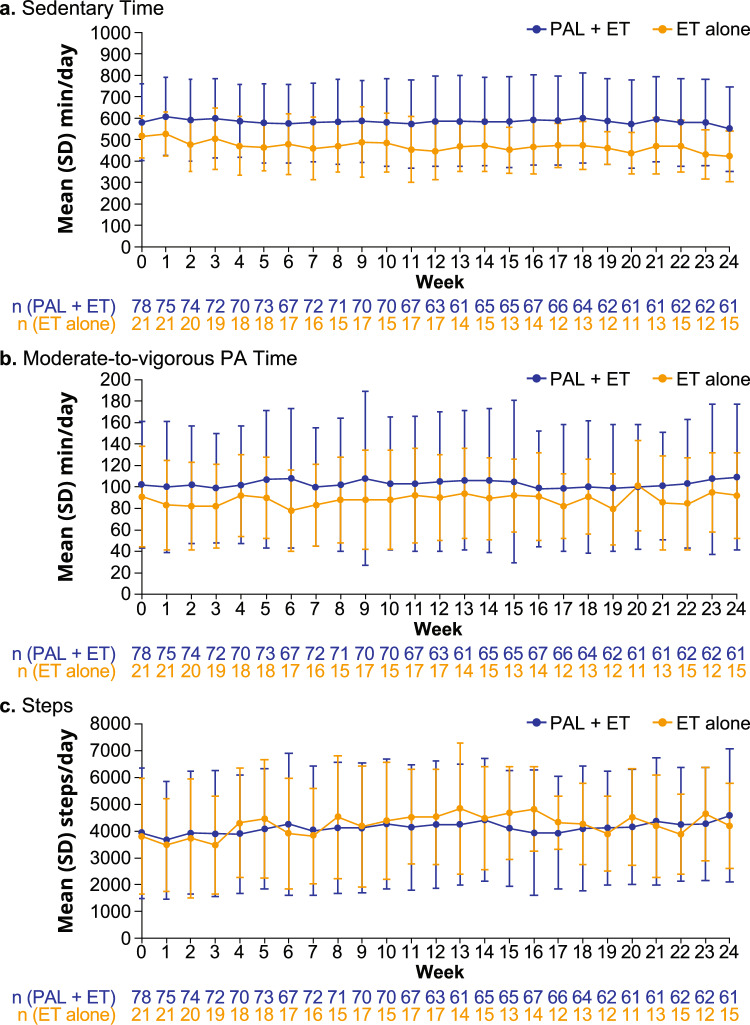
PA metrics by week: **a** sedentary time, **b** moderate-to-vigorous PA time, and **c** steps. *ET* Endocrine therapy, *PA* Physical activity, *PAL* Palbociclib, *SD* Standard deviation

While mean PA metrics differed between the 2 treatment groups (palbociclib plus ET; ET alone) at each time point, the overall trends were similar over the 24-week observation period with no substantial changes observed within groups for sedentary time, moderate-to-vigorous PA time (mean change: 3 min/day;−4 min/day), steps (mean change: 447 steps/day; 202 steps/day) (Fig. 4), light PA time (mean change: −36 min/day;−76 min/day), and calories (mean change: −5 cal/day; 4 cal/day) (Online Resource Fig. 2).

### Safety

Common AEs (all grades) with palbociclib plus ET versus ET alone were neutropenia (70.5% vs 0%), leukopenia (21.8% vs 0%), and anemia (15.4% vs 4.8%). Grade 3/4 neutropenia occurred in 66.7% of patients who received palbociclib plus ET (Table 2). No new safety signals were identified in this study.

**Table 2:** All-cause AEs occurring in > 5% (any grade) of patients or ≥ 1 patient (grade ≥ 3) in the palbociclib plus ET group

AEs, n (%)	Palbociclib + ET (n = 78)		ET alone (n = 21)	
	All grades	Grade ≥ 3	All grades	Grade ≥ 3
Neutropenia	55 (70.5)	52 (66.7)	0	0
Leukopenia	17 (21.8)	10 (12.8)	0	0
Anemia	12 (15.4)	2 (2.6)	1 (4.8)	0
Stomatitis	11 (14.1)	0	0	0
Fatigue	7 (9.0)	1 (1.3)	2 (9.5)	0
AST increased	7 (9.0)	4 (5.1)	1 (4.8)	0
Thrombocytopenia	7 (9.0)	2 (2.6)	1 (4.8)	0
ALT increased	6 (7.7)	5 (6.4)	0	0
Headache	4 (5.1)	0	1 (4.8)	0
Diarrhea	4 (5.1)	0	0	0
COVID-19	2 (2.6)	1 (1.3)	1 (4.8)	1 (4.8)
Febrile neutropenia	1 (1.3)	1 (1.3)	0	0
Death	1 (1.3)	1 (1.3)	0	0
Pneumonia bacterial	1 (1.3)	1 (1.3)	0	0
Blood bilirubin increased	1 (1.3)	1 (1.3)	0	0
Blood LDH increased	1 (1.3)	1 (1.3)	0	0
GGT increased	1 (1.3)	1 (1.3)	0	0

*AE* Adverse event, *ALT* Alanine aminotransferase, *AST* Aspartate aminotransferase, *COVID-19* Coronavirus disease 2019, *ET* Endocrine therapy, *GGT* Gamma-glutamyl transferase, *LDH* Lactate dehydrogenase

Of the 78 patients who received palbociclib plus ET, 56% and 74% had ≥ 1 dose reduction and cycle delay, respectively, over the observation period.

## Discussion

In this real-world study of women with HR +/HER2 − ABC in Japan, neither palbociclib plus ET nor ET alone had any substantial detrimental impacts on HRQOL, based on patient assessment using the ePRO system, and PA, as measured by a wearable device. This is the first real-world study to assess the impact of palbociclib plus ET or ET alone on both HRQOL and PA metrics in women with HR +/HER2 − ABC in Japan. Although our study duration was limited to 24 weeks, the fact that palbociclib plus ET or ET alone did not lead to detriments in HRQOL is notable considering HRQOL scores, measured with the EORTC QLQ-C30 or similar tools, and PA typically decline with disease progression and/or treatment in patients with BC [30, 31]. Our study findings are consistent with those of the PALOMA clinical trials [17, 18], and add to the existing evidence that CDK4/6 inhibitors, including palbociclib, in combination with ET maintain HRQOL among patients with ABC as reported in a systematic review of 31 clinical trials and 7 real-world studies [22].

Among the patients who received palbociclib plus ET, GHS scores averaged 60.9 prior to treatment initiation and 66.1 in the 6th cycle of treatment. Among those patients who received ET alone, GHS scores averaged 64.3 and 63.7, respectively. These GHS scores are similar to those reported in PALOMA-3, in which post-treatment GHS score was to some extent higher in patients who received palbociclib plus fulvestrant versus those who received placebo plus fulvestrant (66.1 vs 63.0; *P* = 0.0313) [18]. Clinical trial HRQOL data are typically captured at Day 1 of a treatment cycle and post-treatment [18]. In this study, PROs were assessed on Day 15 of 6 treatment cycles, thereby providing real-world evidence of patients’ perspectives of their HRQOL while on treatment with palbociclib plus ET.

Guidelines/policies of the European School of Oncology and European Society for Medical Oncology, the ABC Global Alliance, United States Centers for Medicare & Medicaid Services Enhancing Oncology Model, and the PRO working group of the Japanese Association of Supportive Care in Cancer have prioritized the incorporation of validated PRO assessments into oncology research and routine patient care [14, 16, 32, 33]. In addition, it is internationally recognized that electronic monitoring of PROs and real-time data collection has great utility for facilitating communication with patients and care teams, decision-making, and early intervention, all of which are aimed at improving patient overall QOL [14, 16, 32]. Basch and colleagues conducted a seminal randomized controlled trial in the United States that showed that electronic symptom reporting among 766 patients with advanced cancers (breast, genitourinary, gynecologic, and lung) improved HRQOL, reduced emergency and hospital visits, increased time on chemotherapy, and prolonged survival [34, 35]. PRO measures of HRQOL represent key secondary outcomes of oncology research and are particularly important among patients with ABC considering they are living longer in many countries throughout the world, including Japan, but still have an incurable disease [13, 36, 37]. Systematic assessments of how patients function and perform daily activities while on palbociclib plus ET is informative of treatment effectiveness, safety, and tolerability from the patient perspective. Such observational, less burdensome patient data collection may be of high value for clinical decision-making and the timely individualization of patient care for ABC.

Another emerging and increasingly important measurement in oncology research is PA, and wearable devices are progressively being chosen for more objective measurement of patients’ PA activity relative to self-reported estimates [19, 38]. In the current study, at baseline, sedentary time averaged 581 min/day (9.7 h/day) among patients who initiated palbociclib plus ET and 513 min/day (8.6 h/day) among those who initiated ET alone. Over the course of 6 cycles of treatment, sedentary time did not considerably change in either treatment group, which was similarly observed for other PA metrics, including moderate-to-vigorous PA, steps, and light PA. In a literature review of oncology clinical trials in which wearable activity monitors were utilized by patients with cancer (n = 41 trials; 65% BC; 63% post-treatment; 37% patients with active cancer, sedentary time ranged 413 to 556 min/day (n = 6 trials) [19]. Thus, the patients included in the current study had generally similar sedentary behavior as other patients with cancer [19]. For comparison of sedentary time with a general population in Japan, among a large study of 36,023 women aged 35 to 69 years (Japan Multi-Institutional Collaborative Cohort Study, 2004–2014), 47.5% spent < 10 h/day sedentary, while 52.5% spent 10 to ≥ 13 h/day sedentary based on patient survey responses [39]. Maintaining or increasing PA may be especially important for patients with BC in Japan, considering women in Japan spend more time sedentary compared with other populations, which increases their susceptibility to BC [39]. The World Health Organization guidelines on PA and sedentary behavior strongly recommend adults (18─64 years of age) should limit the amount of time spent being sedentary and replace this time with PA of any intensity to yield health benefits [40].

In a meta-analysis that evaluated the association of PA with mortality and the influence of intensity, moderate and high intensity PA post-diagnosis versus low amount of PA, were associated with 31% and 42% significant reductions, respectively, in BC-specific mortality, while decreased PA post-diagnosis was associated with the greatest mortality risk [21]. In a feasibility, single-arm clinical trial of patients with ABC treated with chemotherapy, hormone therapy, or targeted therapy (Advanced stage Breast cancer and Lifestyle Exercise [ABLE]; n = 49) conducted in France, a personalized PA program that included monitoring with a wearable device was found to improve physical outcomes (eg, decreased sitting time, increased 6 min walking distance), while fatigue decreased by 16%, although this was not statistically significant; GHS and functional domain scores of the EORTC QLQ-C30 QOL remained stable [41]. Although not in the scope of the current study, it will be important future research evaluates whether interventions to increase PA among patients with ABC treated with palbociclib plus ET improves HRQOL and prolongs survival.

The findings of this study should be interpreted in the context of its strengths and limitations. The real-world observational design of this study had potential for missing data, which may have been more apparent with the measurement of ePROs. However, ePRO assessments were done repeatedly on Day 15 throughout 6 cycles of treatment, with 95% of patients completing the 6-cycle observation period. Therefore, the data may be more comprehensive than in studies in which such measurements are only completed at the beginning and end of treatment. Also, compliance with ePRO assessment completion was relatively high in the study groups. In this study, PA was measured objectively and longitudinally in real-time with a wearable device and the PA metric data may be more dependable than self-reported survey estimates, which may under- or over-estimate PA metrics; additionally recall bias was avoided [19, 39]. Moreover, PA metric data were collected weekly for 24 weeks, thereby providing relatively continuous estimates of multiple PA types over the course of treatment for ABC.

For this study, ePRO data were captured via a smartphone-based ePRO system, and participating patients must have had some experience with this technology. Those patients with less technological mobile device skills were likely underrepresented. Furthermore, the patients enrolled were willing to complete data collection and wear the wearable device. They also had an ECOG PS of 0 or 1. Thus, the patients may represent a more knowledgeable, engaged and healthier set of patients. This study had a small sample size, the observation period was 24 weeks, and only women from Japan were included. Patient selection, treatments received, and monitoring procedures were based on the discretion of the treating physician in routine clinical practice; thus, there is a potential for selection and responder bias. Between- and within-group comparisons were not performed due to the study design, which was not aimed to show superiority or inferiority, and did not have a sample size calculation. Only associations, not causality, can be inferred between treatments and outcomes due to the observational nature of this study. Lastly, the observational period may have resulted in a seasonal effect on PA. Despite the seasonal variations, the pace of patient enrollment remained relatively constant.

## Conclusions

In this real-world study of women with HR +/HER2 − ABC in Japan, neither palbociclib plus ET nor ET alone had any substantial detrimental impacts on HRQOL, as measured by the ePRO system, and PA, as measured with a wearable device. Future studies are needed to identify barriers to use of ePRO systems and wearable devices, including not only technological skill level, but also sociodemographic characteristics and other patient factors, so that they can be mitigated with support services and patient care can be delivered equitably.

## Supplementary Information

Below is the link to the electronic supplementary material.

Supplementary file1 (DOCX 927 KB)

## Data Availability

Upon request, and subject to review, Pfizer will provide the data that support the findings of this study. Subject to certain criteria, conditions and exceptions, Pfizer may also provide access to the related individual de-identified participant data. See https://www.pfizer.com/science/clinical-trials/trial-data-and-results for more information.
