# Supplementary material for: Health-related quality of life and physical activity collected via mobile application and wearable device in patients with HR +/HER2 − advanced breast cancer treated with palbociclib plus endocrine therapy or endocrine therapy alone: 6-month longitudinal study (JBCRG-26)
Source: Breast Cancer. 2025 Jul 18;32(5):1132–43. doi: 10.1007/s12282-025-01744-0 (PMC12394391; doi:10.1007/s12282-025-01744-0)
Supplement: Supplementary file 1 — Supplementary file1 (DOCX 927 KB) [file 12282_2025_1744_MOESM1_ESM.docx]

**Supplementary material**

**Health-related quality of life and physical activity collected via mobile application and wearable device in patients with HR+/HER2− advanced breast cancer treated with palbociclib plus endocrine therapy or endocrine therapy alone: 6-month longitudinal study (JBCRG-26)**

**Journal:** Breast Cancer

Hiroko Bando,^1^ Aya Ueda,^2^ Kaori Terata,^3^ Mihoko Doi,^4^ Shigenori E. Nagai,^5^ Masaya Hattori,^6^ Kenichi Watanabe,^7^ Nobuko Tamura,^8^ Manabu Futamura,^9^ Kei Koizumi,^10^ Naoki Niikura,^11^ Tempei Miyaji,^12^ Yasuaki Muramatsu,^13^ Linghua Xu,^14^ Norikazu Masuda,^15^ Shigehira Saji^16^

**Corresponding author:** Hiroko Bando

Department of Breast-Thyroid-Endocrine Surgery

Institute of Medicine, University of Tsukuba

Tsukuba, Japan

Email: [bando@md.tsukuba.ac.jp](mailto:bando@md.tsukuba.ac.jp)

ORCID: 0000-0002-7361-3647

**Online Resource Fig. 1 Mean (SD) scores of EORTC QLQ-C30 symptom subscales**


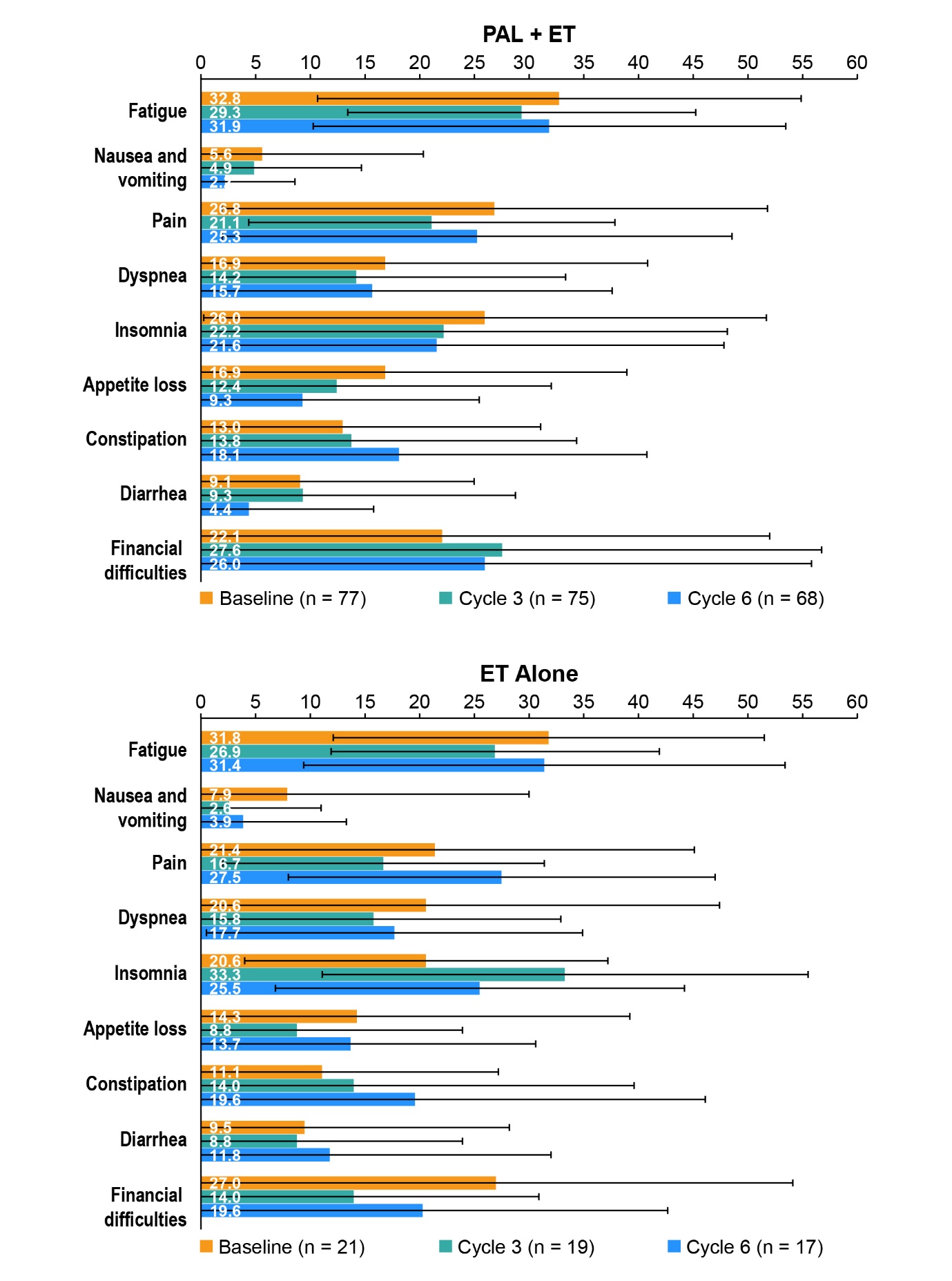


EORTC QLQ-C30, European Organisation for Research and Treatment of Cancer Quality-of-Life Questionnaire-C30; ET, endocrine therapy; PAL, palbociclib; SD, standard deviation.

Higher scores indicate greater symptom severity.

**Online Resource Fig. 2 PA metrics by week: a) wear time, b) light PA time and c) calories**


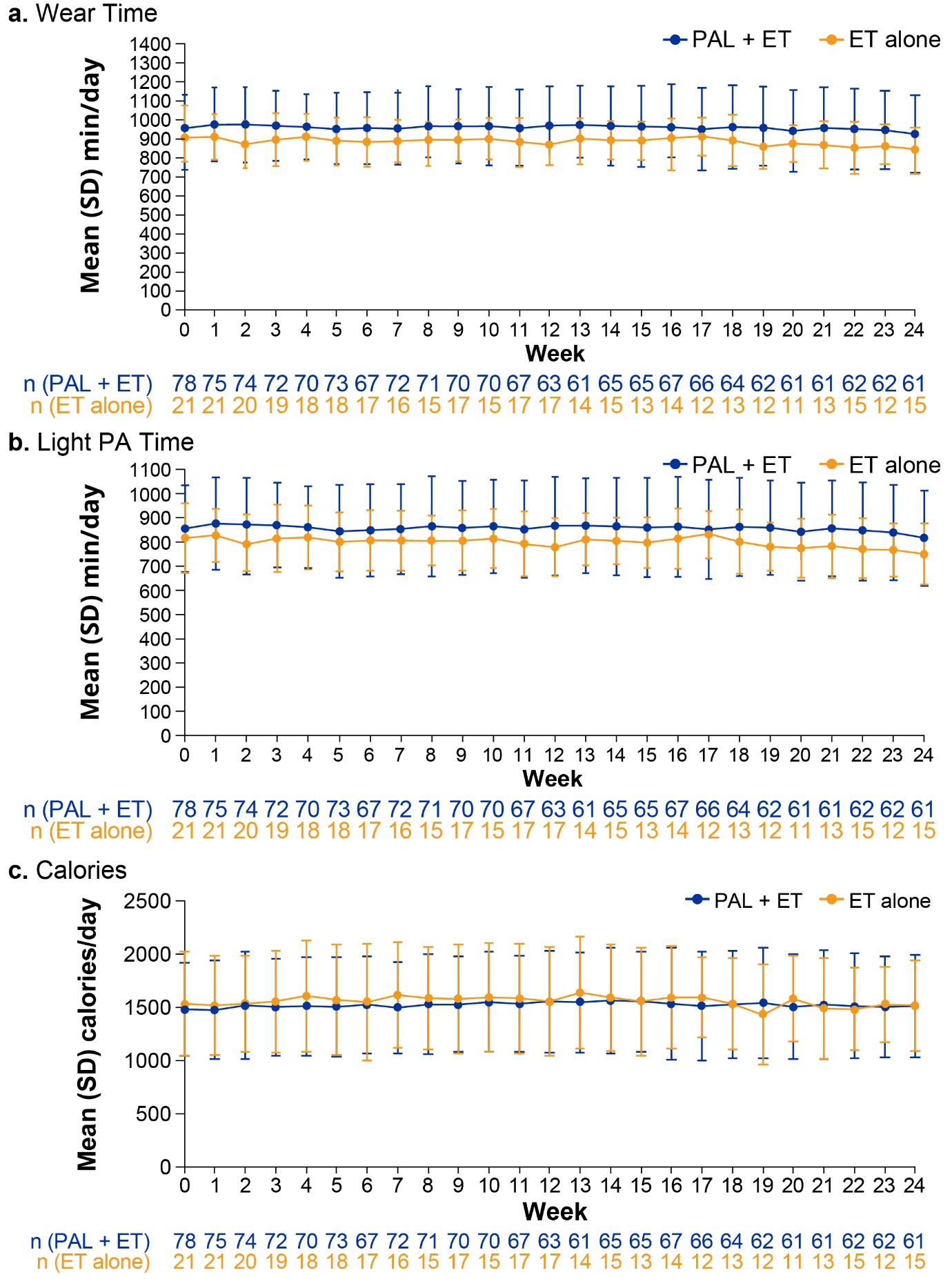


ET, endocrine therapy; PA, physical activity; PAL, palbociclib; SD, standard deviation.
